# Lineage^−^CD34^+^CD31^+^ Cells That Appear in Association with Severe Burn Injury Are Inhibitory on the Production of Antimicrobial Peptides by Epidermal Keratinocytes

**DOI:** 10.1371/journal.pone.0082926

**Published:** 2014-02-03

**Authors:** Shohei Yoshida, Jong O. Lee, Kiwamu Nakamura, Sumihiro Suzuki, David N. Hendon, Makiko Kobayashi, Fujio Suzuki

**Affiliations:** 1 Department of Internal Medicine, The University of Texas Medical Branch, Galveston, Texas, United States of America; 2 Shriners Hospital for Children, Galveston, Texas, United States of America; 3 Department of Biostatistics, University of North Texas Health Science Center, Fort Worth, Texas, United States of America; University of Cincinnati, United States of America

## Abstract

Antimicrobial peptides are major host defense effectors against *Pseudomonas aeruginosa* skin infections. Due to the lack of such peptide production, severely burned hosts are greatly susceptible to *P. aeruginosa* burn wound infection. β-Defensin (HBD) production by normal human epidermal keratinocytes (NHEK) was inhibited by lineage^−^CD34^+^ cells isolated from peripheral blood of severely burned patients. Lineage^−^CD34^+^ cells obtained from severely burned patients were characterized as CD31^+^, while healthy donor lineage^−^CD34^+^ cells were shown to be CD31^−^ cells. Lineage^−^CD34^+^CD31^−^ cells did not show any inhibitory activities on HBD-1 production by NHEK. CCL2 and IL-10 released from lineage^−^CD34^+^CD31^+^ cells were shown to be inhibitory on the peptide production by NHEK, while these soluble factors were not produced by lineage^−^CD34^+^CD31^−^ cells. After treatment with a mixture of mAbs for CCL2 and IL-10, the culture fluids of lineage^−^CD34^+^CD31^+^ cells did not show any inhibitory activities on HBD-1 production by NHEK. Lineage^−^CD34^+^CD31^+^ cells that appear in association with burn injuries play a role on the inhibition of antimicrobial peptide production by skin keratinocytes through the production of CCL2 and IL-10.

## Introduction

Burn patients are particularly susceptible to various infections [1]–[3]. In these patients, burn wound infections can easily escalate into sepsis [4]. *Pseudomonas aeruginosa* is a pathogen most frequently isolated from severely burned patients, and a major source for life-threatening infections in these patients [5]. Normally, topical antimicrobials are effective for controlling the colonization and multiplication of the pathogen on the surface of burn wounds [4]; however, due to the burn-associated defects of a host's antimicrobial innate immunity, the small numbers of *P. aeruginosa* that elude treatment are sufficient to spread systemically [6]. Skin is recognized as a physiological barrier against microorganisms and functions as a host's defense mechanism against invading pathogens by producing antimicrobial peptides [7]–[9]. Antimicrobial peptides produced by skin keratinocytes kill the pathogen by adhering to and punching a hole in the fatty cell membranes of the bacteria and chemoattract various immunocompetent cells to the infection sites [7]–[9]. The lack of antimicrobial peptides in the skin allows surface growth and subsequent spreading of the pathogens throughout the body [10]–[13]. Some antimicrobial peptides are synthesized naturally, whereas some are produced after exposure to microbes [7]–[9]. β-Defensins, mainly produced by epidermal keratinocytes, are one of the major families of skin antimicrobial peptides. Gene-defect experimental models and engineered epidermis have explored the importance of β-defensins in controlling skin infections [10]–[13]. However, the lack of β-defensins in burned skin and in burn blister fluids has been described [14], [15]. In our previous studies, the homogenates of tissues surrounding the burn area did not contain sufficient amounts of antimicrobial peptides [16]. Furthermore, normal mice treated with anti-murine skin antimicrobial peptide IgG did not resist skin infection with 100 CFU/mouse of *P. aeruginosa* [17]. These results indicate that the lack of antimicrobial peptide production in tissues surrounding the burn sites is a key factor for the development of sepsis caused by *P. aeruginosa* burn wound infections.

In previous murine studies 16,18, we have demonstrated that cells isolated from tissues surrounding the burn sites were inhibitory on the production of antimicrobial peptides by epidermal keratinocytes, and that these inhibitor cells were characterized as Gr-1^+^CD11b^+^CD34^+^ CD40^+^ cells [16]. Similar to the murine studies, we recently isolated CD34^+^ cells from a hematopoietic lineage cell-depleted preparation (lineage^−^CD34^+^ cells) of peripheral blood cells from severely burned patients; these cells were shown to be inhibitory on the production of antimicrobial peptides by normal human epidermal keratinocytes (NHEK). In other studies, the suppressor cell activity of bone-marrow-derived lineage^−^CD34^+^ cells against T cell blastformation has been reported [19]. Also, an increase in the number of lineage^−^CD34^+^ leukemia stem cells in cancer patients has been reported, where these cells are considered to be involved in tumor-associated immunosuppression [20]–[22]. Therefore, in this paper, burn patient lineage^−^CD34^+^ cells were further characterized for their inhibitory functions on the antimicrobial peptide production by NHEK.

In this study, lineage^−^CD34^+^ cells isolated from the peripheral blood of severely burned patients were characterized as CD31^+^ cells, which are known to be inhibitory on HBD-1 production by NHEK. CCL2 and IL-10 released from burn patient lineage^−^CD34^+^CD31^−^ cells were shown to be responsible for their inhibitory activities on the production of HBD-1 by NHEK.

## Materials and Methods

### Ethics statement

The study was approved by the Institutional Review Board of the University of Texas Medical Branch (IRB approved number: 02-018). A written informed consent for blood sampling was obtained from all adult subjects. For blood sampling from children, written parental consent was obtained. Ethical approval was obtained from the Ethical and Scientific Committee of the University of Texas Medical Branch.

### Thermally injured patients

Twenty severely burned patients (14 male, 6 female) admitted to the Shriners Hospitals for Children at Galveston and the University of Texas Medical Branch (UTMB) were enrolled in this study (**Table 1**). All patients had more than a 30% total body surface area (TBSA) burn (average 55.7±18.3%). The youngest and oldest ages were 4 and 54 years old (average 15.2±14.7), respectively. All patients were subjected to a standard treatment regimen [23]. This regimen consisted of fluid resuscitation, topical antimicrobial agents, and aggressive nutrition support based on estimated energy and protein needed.

**Table 1:** Burn patients and healthy donors enrolled in this study.

Patients* (#)	Sex	Age (y)	TBSA burn (%)	Post burn days
1–7 days post burn				
1	M	29	56	4
2	M	33	30	4
3	M	54	38	0
4	M	5	44	3
5	M	4	36	3
6	M	9	75	6
7	M	8	70	1
8	M	8	81	1
9	M	13	65	4
1–4 weeks post burn				
10	M	11	35	14
11	M	9	75	16
12	M	10	46	14
13	F	50	89	24
14	F	4	40	17
15	F	8	40	10
1–3 years post burn				
16	F	9	65	829
17	M	4	84	448
18	M	8	44	586
19	F	12	49	212
20	M	16	53	697
Healthy donors (#)				
1	M	37		
2	F	4		
3	M	38		
4	M	30		
5	F	9		

* All patients were admitted to the Shriners Hospitals for Children at Galveston from February 2008 to July 2011.

### Reagents, media, and cells

Human β-defensin-1 (HBD-1) kits were purchased from PeproTech (Rocky Hill, NJ, USA). HBD-1, murine β-defensin-1 (MBD-1), anti-HBD-1, and anti-MBD-1 antibodies were purchased from Alpha Diagnostic International (San Antonio, TX, USA). Lineage cell depletion kits were purchased from Miltenyi Biotec (Auburn, CA, USA). FITC-conjugated anti-CD34 mAb and PE-conjugated anti-CD31 mAb were purchased from BD Biosciences (Franklin Lakes, NJ, USA) and BioLegend (San Diego, CA, USA), respectively. Recombinant (r) CCL2, rIL-4, rIL-10, and rIL-13 were obtained from PeproTech. mAbs directed against CCL2, IL-10, and IL-13 were purchased from BioLegend. Adult normal human epidermal keratinocytes (NHEK) were obtained from Lonza (Walkersville, MD, USA) and propagated in a serum-free keratinocyte growth medium (KMG-2, Lonza) at 37°C. NHEK that underwent a second passage with KMG-2 were stored in liquid nitrogen. NHEK grown from the stored cells (the third passage) were used in this study. RPMI-1640 medium supplemented with 10% FBS, 2 mM L-glutamine, and antibiotics (100 U/ml penicillin and 100 µg/ml streptomycin) was used as culture media for lineage^−^CD34^+^ cells.

### Preparation of lineage^−^CD34^+^ cells

Lineage^−^CD34^+^ cells were prepared from peripheral blood mononuclear cells (PBMC) of thermally injured patients and healthy donors. Because the amount of blood specimens from each burn patient was not enough for all assays, we have developed each experiment utilizing available patient blood as follows: RT-PCR analysis (burn patients #1, #16, #17, #18 and #19), flow cytometric analysis (burn patients #4, #5, #6, #11, #12) and analysis of culture fluids (burn patients #8, #9, #13, #14 and #15). PBMC were isolated from heparinized whole blood cells using Ficoll-Hypaque sedimentation [24], [25]. Mature hematopoietic cells (T cells, B cells, NK cells, dendritic cells, monocytes and granulocytes) were magnetically depleted from the PBMC using a lineage cell depletion kit. Then, CD34^+^ cells were sorted from these cells by a FACSAria flow cytometer (Becton Dickinson) with FITC-conjugated anti-CD34 mAb. In some experiments, CD31^+^ cells were further isolated from burn patient lineage^−^CD34^+^ cell preparations by a FACSAria cell sorter with PE-conjugated anti-human CD31 mAb.

### In vitro assays of lineage^−^CD34^+^ cells

Lineage^−^CD34^+^ cells were tested for their abilities to inhibit HBD-1 production by NHEK. Thus, NHEK (1×10^5^ cells/ml, lower chamber) were cultured with lineage^−^CD34^+^ cells (upper chamber) in a dual-chamber transwell (0.4 µm pore size, Costar, Corning, NY, USA). Thirty-six hours after cultivation, the upper chamber was removed and NHEK in the lower chamber were cultured for an additional 36 hours. Culture fluids obtained were assayed for HBD-1 by ELISA, and cells obtained were analyzed for HBD-1 mRNA by semi-quantitative RT-PCR. Total RNA was extracted from these NHEK and turned back into cDNA through the reverse transcription of mRNA. β-actin was utilized as an internal control to normalize sample to sample variations in total RNA amounts. PCR was carried out using synthesized oligonucleotide primers: HBD-1, 5′–CCCAGTTCCTGAAATCCTGA–3′ (F) and 5′–CTTCTG GTCACTCCCAGCTC–3′ (R). Using a thermal cycler, 35 cycles of PCR were performed at 94°C for 30 seconds, 61°C for 30 seconds, and 72°C for 30 seconds. The predicted products were run on 2% agarose gels containing ethidium bromide, and densitometric analysis was performed using a UVP Bioimaging System. To determine CD31 expression, lineage^−^CD34^+^ cell preparations from burn patients and healthy donors were stained with PE-conjugated anti-human CD31 mAb. Fluorescence-positive cells were analyzed by FACS Canto (Becton-Dickinson) and FlowJo software.

### Preparation of lineage^−^CD34^+^CD31^+^ cell-culture fluids and assay for their inhibitory activities on HBD production from NHEK

To determine the mechanism involved in the inhibitory activity of lineage^−^CD34^+^CD31^+^ cells on HBD production from NHEK, lineage^−^CD34^+^CD31^+^ cells (1×10^6^ cells/ml) were cultured for 12 to 48 hours without any stimulation. As a control, lineage^−^CD34^+^CD31^−^ cells derived from healthy donors were cultured under the same conditions. The culture fluids harvested were assayed for various cytokines and chemokines using ELISA. IL-4, IL-10, IL-12, IL-13, IL-17, IL-18, IL-23, IFN-γ, CCL1, CCL2, CCL3, and CCL5 were included in the assay. The minimum detection limit for these cytokines and chemokines was 2–15 pg/ml. Next, the culture fluids of lineage^−^CD34^+^CD31^+^ cells were treated with 2.5 µg/ml of mAbs against soluble factors detected in the conditioned media for 1.5 hours at 4°C to determine the effector(s) for inhibiting HBD-1 production. As a control, the same culture fluids were treated with isotype Ab in the same fashion. Then, each culture fluid (20% v/v) was added to cultures of NHEK (1×10^6^ cells/ml). In some experiments, NHEK were cultured with media supplemented with recombinant cytokines or chemokines detected in the culture fluids of lineage^−^CD34^+^CD31^+^ cells. NHEK cultured with media alone served as a control. Culture fluids were harvested 36 hours after the cultivation and assayed for HBD-1 by ELISA. The minimum detection limit for HBD-1 was 4 pg/ml.

### Statistical analyses

Data are presented as mean ± SEM. The results of the test group were compared to those of the control group using a Student's *t* test. If the *P*-value was less than 0.05, the result was considered to be significant.

## Results

### Lineage^−^CD34^+^ cells from severely burned patients are inhibitory on HBD-1 production by NHEK

**Figure 1:**
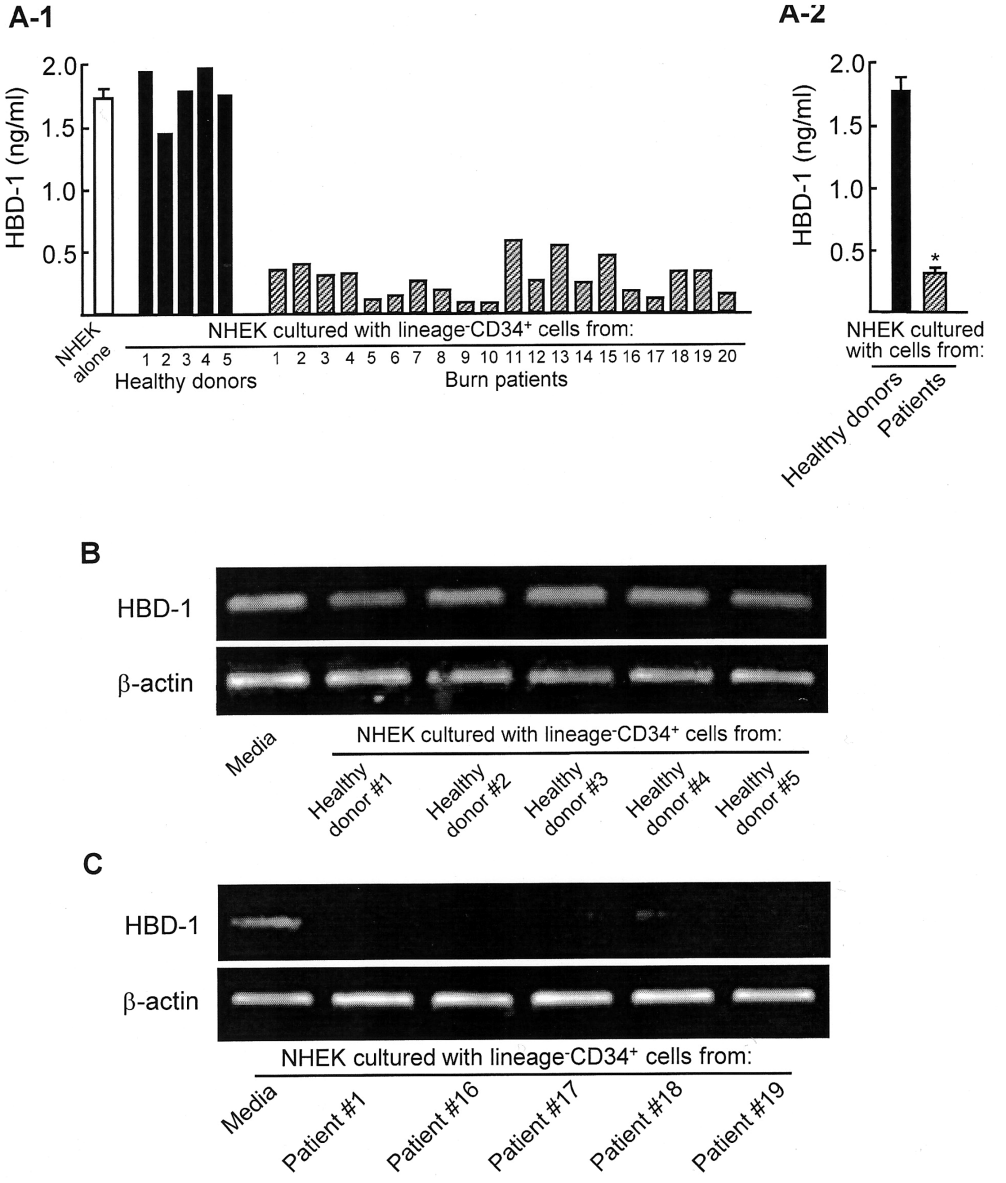
HBD-1 production and mRNA expression by NHEK cultured with peripheral blood lineage^−^CD34^+^ cells that were isolated from both severely burned patients and healthy donors. A. HBD-1 production. Lineage^−^CD34^+^ cells (1×10^5^ cells/ml, upper chamber), isolated from 5 healthy donors (#1∼#5) and 20 burn patients (#1∼#20), were transwell-cultured with NHEK (1×10^5^ cells/ml, lower chamber) for 36 hours. After removal of the upper chamber, NHEK in the lower chamber were cultured for an additional 36 hours. Culture fluids obtained were assayed for HBD-1 by ELISA. Fig. 1A-1 shows independent experiments performed using blood specimens from 5 healthy donors and 20 burn patients, and Fig. 1A-2 shows mean ± SEM of the results shown in Fig. 1A-1. * *P*<0.001 vs control. **B and C.** HBD-1 mRNA expression. Lineage^−^CD34^+^ cells (1×10^5^ cells/ml, upper chamber), isolated from peripheral blood of 5 healthy donors (#1∼#5, **B**) and 5 severely burn patients (#1, #16, #17, #18, #19, **C**), were transwell-cultured with NHEK (1×10^5^ cells/ml, lower chamber) for 36 hours. After removal of the upper chamber, NHEK in the lower chamber were analyzed for HBD-1 mRNA by RT-PCR.

The effect of burn patient lineage^−^CD34^+^ cell populations on β-defensin-1 (HBD-1) production and mRNA expression by NHEK was examined. All 20 burn patients enrolled in this study are listed in **Table 1**. In the first series of experiments, lineage^−^CD34^+^ cells (1×10^5^ cells/ml, upper chamber) were isolated from PBMC of 5 healthy donors and 20 severely burned patients and transwell-cultured with NHEK (1×10^5^ cells/ml, lower chamber) for 36 hours. After removal of the upper chamber, NHEK in the lower chamber were cultured for an additional 36 hours, and culture fluids harvested were assayed for HBD-1 by ELISA. In the results, 1.6 to 1.9 ng/ml of HBD-1 were produced by NHEK transwell-cultured with healthy donor lineage^−^CD34^+^ cells. However, HBD-1 production by NHEK was suppressed by 62∼94% when these cells were transwell-cultured with burn patient lineage^−^CD34^+^ cells (**Fig. 1a**). Similar results were obtained when HBD-1 mRNA expression by NHEK harvested from the lower chamber of transwells was assayed by RT-PCR. As controls, HBD-1 mRNA was consistently expressed by NHEK transwell-cultured with #1∼#5 healthy donor lineage^−^CD34^+^ cells (**Fig. 1b**). However, HBD-1 mRNA was not expressed by NHEK transwell-cultured with lineage^−^CD34^+^ cells derived from burn patients (**Fig. 1c**).

#### CD31 is expressed by burn patient lineage^−^CD34^+^ cells

**Figure 2:**
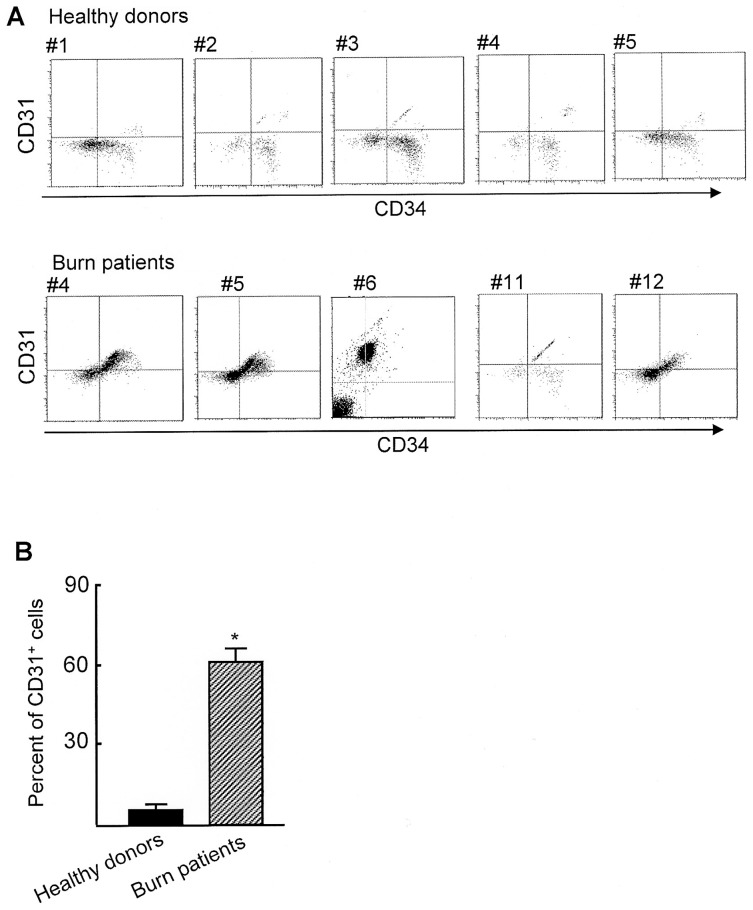
CD31^+^ cells detected in burn patient peripheral blood lineage^−^CD34^+^ cells. Lineage^−^CD34^+^ cells from healthy donors and burn patients were analyzed for CD31 expression by flow cytometry. Fig. 2A shows independent experiments performed using blood specimens from 5 healthy donors and 5 burn patients (#4, #5, #6, #11, and #12), and Fig. 2B shows mean ± SEM of the results shown in Fig. 2A. * *P*<0.001 vs control.

Lineage^−^CD34^+^ cells were shown to be inhibitory on HBD-1 production by NHEK, while the peptide production by NHEK was not influenced by the same cell preparations derived from healthy donors. Therefore, peripheral blood lineage^−^CD34^+^ cells isolated from severely burned patients and healthy donors were analyzed for CD31 surface antigen expression by flow cytometry. The expression of the CD31 surface antigen by CD4^+^ suppressor cells and myeloid-derived suppressor cells has been demonstrated [26]–[28]. **Figs. 2a and 2b** show the results of flow cytometric assay with CD34 and CD31 double staining analyzed for all 5 healthy donors and 5 randomly selected burn patients (#4, #5, #6, #11 and #12). In the burn patient lineage^−^CD34^+^ cell preparations, 60% or more were shown to be CD31^+^ cells. In contrast, only 0.2 to 8.2% in the healthy donor lineage^−^CD34^+^ cell preparations were CD31^+^ cells.

### Mechanisms involved in the suppressor cell activity of lineage^−^CD34^+^CD31^+^ cells on HBD-1 production

**Figure 3:**
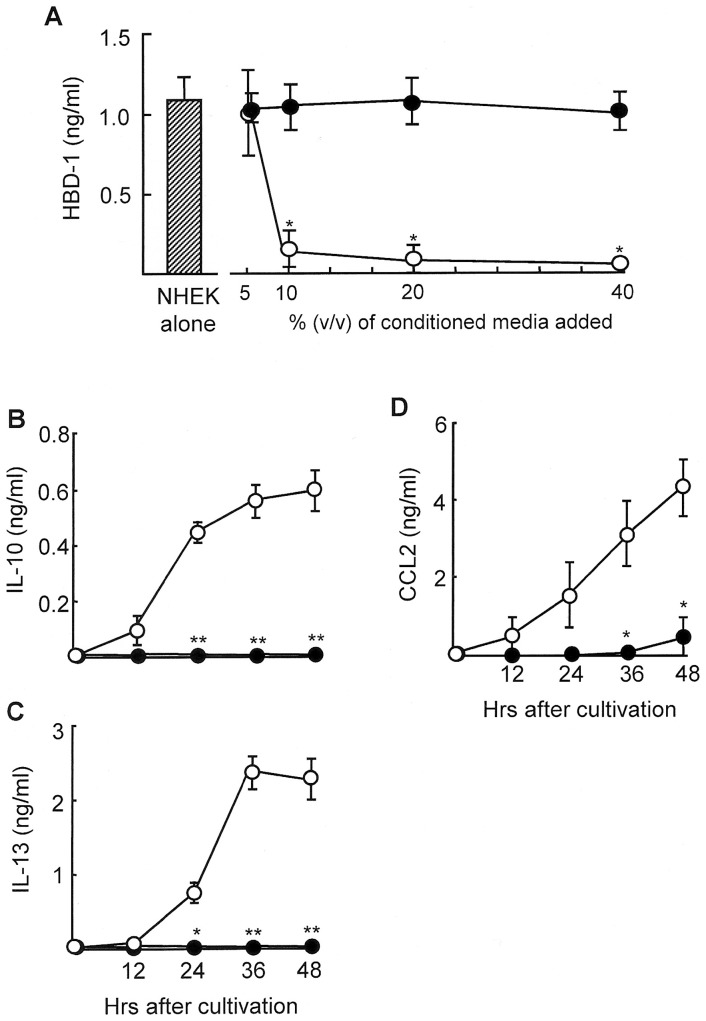
Culture fluids of lineage^−^CD34^+^CD31^+^ cells are inhibitory on HBD-1 production by NHEK. A. Inhibition of HBD production in NHEK cultures supplemented with culture fluids of lineage^−^CD34^+^CD31^+^ cells. Culture fluids were harvested 48 hours after cultivation of lineage^−^CD34^+^CD31^+^ cells (1×10^6^ cells/ml) derived from burn patients #8, #9, #13, #14 and #15 (open circles). The culture fluids of lineage^−^CD34^+^CD31^−^ cells of healthy donors #1∼#5 (filled circles) were utilized as a control. Five to 40% (v/v) of these culture fluids were added to cultures of NHEK (1×10^5^ cells/ml). Culture fluids harvested from NHEK cultures were assayed for HBD-1 by ELISA. * *P*<0.01 vs control. **B–D.** Production of IL-10, IL-13 and CCL2 by lineage^−^CD34^+^CD31^+^ cells. Lineage^−^CD34^+^CD31^+^ cells (1×10^5^ cells/ml, open circles) isolated from peripheral blood of burn patients #8, #9, #13, #14 and #15 were cultured for 12 to 48 hours. As controls, peripheral blood lineage^−^CD34^+^CD31^−^ cells of healthy donors #1∼#5 (filled circles) were cultured under the same conditions. Supernatants obtained were assayed for IL-10 (**B**), IL-13 (**C**) and CCL2 (**D**) by ELISA. * *P*<0.05, ** *P*<0.01 vs lineage^−^CD34^+^CD31^−^ cells.

As shown in **Fig. 1**, burn patient lineage^−^CD34^+^ cells inhibited HBD-1 production by NHEK in transwell-cultures. This indicates that some soluble factors released from lineage^−^CD34^+^CD31^+^ cells are involved in the inhibition of HBD-1 production by NHEK. Therefore, NHEK (1×10^5^ cells/ml) were cultured in fresh medium (RPMI-1640/10% FBS) supplemented with various volumes (5–40%, v/v) of culture fluids of lineage^−^CD34^+^CD31^+^ cells. The culture fluids were harvested from 1×10^6^ cells/ml of the cultures of burn patient lineage^−^CD34^+^CD31^+^ cells 48 hours after cultivation. When 10% (v/v) of the culture fluids were added to NHEK cultures, 86% of the HBD-1 production was reduced. More than 96% inhibition was demonstrated when 20% (v/v) or more of the culture fluids were added to NHEK cultures (**Fig. 3a**). However, amounts of HBD-1 produced by NHEK were not influenced when these cells were cultured with 10 to 40% (v/v) of the culture fluids derived from cultures of healthy donor lineage^−^CD34^+^CD31^−^ cells. From these results, the culture fluids of lineage^−^CD34^+^CD31^+^ cells were shown to be inhibitory on HBD-1 production by NHEK.

To determine what soluble factors are present in the culture fluids of lineage^−^CD34^+^CD31^+^ cells, the culture fluids of these cells were randomly assayed for IL-4, IL-10, IL-12, IL-13, IL-17, IL-18, IL-23, IFN-γ, CCL1, CCL2, CCL3, and CCL5. As a result, IL-10, IL-13, and CCL2 were detected in the culture fluids of lineage^−^CD34^+^CD31^+^ cells. Other soluble mediators were not demonstrated in the culture fluids of these cells. The time course of the production of IL-10, IL-13, and CCL2 is shown in **Figs. 3b–3d**. Lineage^−^CD34^+^CD31^−^ cells derived from peripheral blood of healthy donors did not produce IL-10, IL-13, or CCL2 (Figs. 3b–3d).

**Figure 4:**
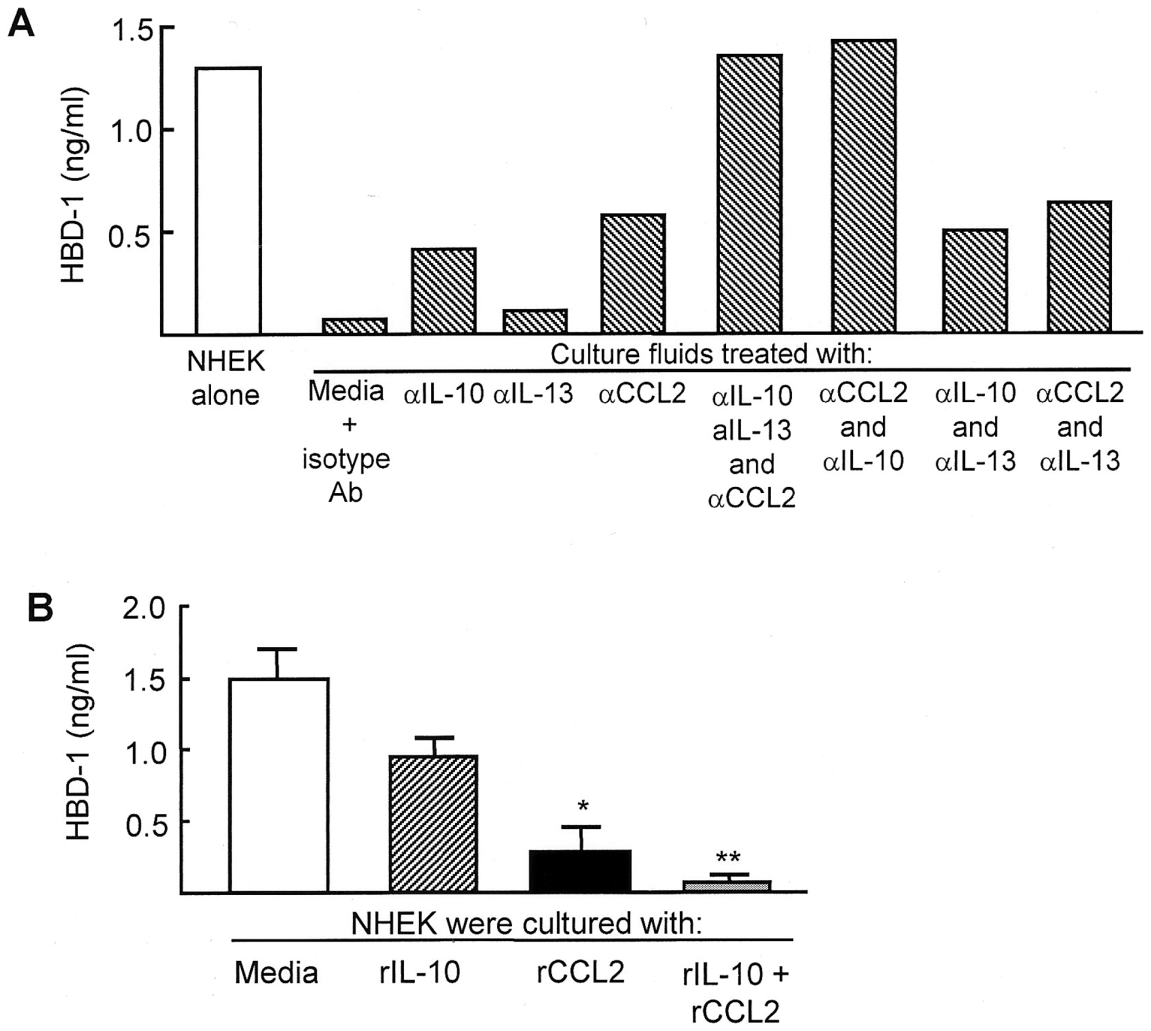
CCL2 and IL-10 produced by burn patient lineage^−^CD34^+^ CD31^+^ cells are responsible for their suppressor cell activities on HBD-1 production by NHEK. A. Effect of mAbs against IL-10, CCL2 and IL-13 on the suppressor activities of lineage^−^CD34^+^CD31^+^ cell-culture fluids on HBD production by NHEK. NHEK (1×10^5^ cells/ml) were cultured with fresh media supplemented with 20% (v/v) of the culture fluids that were previously treated with mAb for IL-10, IL-13 and CCL2 (2.5 µg/ml each) or a combination of these mAbs. Forty-eight hours after cultivation, culture fluids harvested were assayed for HBD-1. Figure 4A displays one of the representative results shown in independent experiments using culture fluids of 5 different lineage^−^CD34^+^CD31^+^ cell preparations derived from burn patients #8, #9, #13, #14 and #15. **B.** Effect of rIL-10 and rCCL2 on HBD-1 production by NHEK. NHEK (1×10^5^ cells/ml) were treated with rIL-10 (1 ng/ml), rCCL2 (10 ng/ml) or a combination of rIL-10 and rCCL2. Culture fluids obtained 24 hours after treatment were assayed for HBD-1. * *P*<0.01, ** *P*<0.001 vs NHEK cultured with media.

Next, the role of IL-10, IL-13, and CCL2 on the lineage^−^CD34^+^CD31^+^ cell-associated suppression of HBD-1 production by NHEK was examined. The culture fluids of lineage^−^CD34^+^CD31^+^ cells were treated with a mAb directed against IL-10, IL-13, and CCL2, or a mixture of these mAbs. Then, these culture fluids were assayed for their inhibitory activities on the peptide production by NHEK. When NHEK were cultured with the lineage^−^CD34^+^CD31^+^ cell-conditioned media that were previously treated for 1.5 hours at 4°C with anti-IL-10 or anti-CCL2 mAb, HBD-1 production was recovered by 33 to 48%. The complete recovery of the HBD-1 production was shown when lineage^−^CD34^+^CD31^+^ cell-conditioned media previously treated with a mixture of mAbs for IL-10 and CCL2 were added to NHEK cultures (**Fig. 4a**). HBD-1 production by NHEK was not influenced significantly (5% recovery) when NHEK were cultured with the conditioned media treated with anti-IL-13 mAb. Any synergistic effects between IL-13 and IL-10 or IL-13 and CCL2 were not demonstrated. These results indicate that IL-10 and CCL2 are effector molecules of lineage^−^CD34^+^CD31^+^ cells for their inhibitory activities on HBD-1 production by NHEK. In fact, a mixture of rIL-10 and rCCL2 completely suppressed HBD-1 production by NHEK (**Fig. 4b**).

## Discussion

HBDs are important effector molecules in the antibacterial innate immunities against dermal infections [10]–[13]. However, expressions of HBD-1 and HBD-2 mRNAs are greatly reduced in tissues surrounding the full-thickness burn wounds [14]–[16]. In the present study, lineage^−^CD34^+^ cells isolated from the peripheral blood of severely burned patients were shown to be inhibitory on HBD-1 production by normal human epidermal keratinocytes (NHEK), while the same lineage^−^CD34^+^ cells from healthy donor peripheral blood did not inhibit the peptide production by NHEK. Suppressor cells in burn patient peripheral blood were characterized as lineage^−^CD34^+^CD31^+^ cells, while healthy donors were shown to be a carrier of lineage^−^CD34^+^CD31^−^ cells. The expression of the CD31 surface antigen by human CD4^+^ suppressor cells has been demonstrated [26]. Murine myeloid-derived suppressor cells have been reported as cells expressing Gr-1, CD11, and CD31 surface antigens [27], [28]. These reports support our findings that lineage^−^CD34^+^CD31^+^ cells demonstrated in the burn patient peripheral blood are suppressor cells for HBD production by NHEK. In our preliminary studies, γ-irradiated NOD-SCID IL-2rγ^−/−^ mice resisted skin infection with 10^3^ CFU/mouse of *P. aeruginosa*. *P. aeruginosa* at a dose of 10^5^ CFU/mouse was shown to be 1 LD_50_ in γ-irradiated NOD-SCID IL-2rγ^−/−^ mice. These mice are deficient for functional T cells, B cells, NK cells, macrophages, and neutrophils [29]–[31], indicating antimicrobial peptides released from non-immunocompetent cells play a role in the protection from sepsis stemming from *P. aeruginosa* skin infections. On the other hand, the same mice treated with anti-mouse skin AMP rabbit IgG died quickly after 10^5^ CFU/mouse of *P. aeruginosa* skin infection. Less than 10 CFU/mouse of *P. aeruginosa* was shown to be 1 LD_50_ in γ-irradiated NOD-SCID IL-2rγ^−/−^ mice treated with the IgG. MBD-1, MBD-2, and MBD-3 (typical murine antimicrobial peptides in skin) were not detected in skin homogenates of γ-irradiated NOD-SCID IL-2rγ^−/−^ mice treated with the IgG. This indicates that skin antimicrobial peptides play an important role in the host's antibacterial innate immunities against *P. aeruginosa* skin infections. This suggests that the host's antibacterial defense against sepsis stemming from pseudomonal burn wound infections is impaired by lineage^−^CD34^+^CD31^+^ cells that appear in association with burn injuries.

Lineage^−^CD34^+^CD31^+^ cells inhibited HBD-1 production by NHEK in dual-chamber transwells. This indicates that cell-to-cell contact between lineage^−^CD34^+^CD31^+^ cells and NHEK was not required for inhibiting HBD-1 production from NHEK. Therefore, we examined the role of soluble factors released from lineage^−^CD34^+^CD31^+^ cells on inhibiting HBD-1 production from NHEK. In the first series of experiments, various cytokines and chemokines in the cultures of lineage^−^CD34^+^CD31^+^ cells were randomly assayed. In the results, IL-10, CCL2, and IL-13 were detected in the culture fluids of lineage^−^CD34^+^CD31^+^ cells. Other soluble factors were not detected in the culture fluids of these cells. IL-13 has been shown to be involved in HBD-3 deficiency in atopic dermatitis [32]–[34]. Expressions of HBD-2 and LL37 by keratinocytes were shown to be down-regulated by IL-10 and/or IL-13 [33]. Also, the importance of IL-4 on inhibiting HBD production has been substantiated [35]; however, IL-4 was not detected in these culture fluids. None of the above cytokines and chemokines were produced by healthy donor lineage^−^CD34^+^CD31^−^ cells, which were not inhibitory on HBD-1 production from NHEK. In the next series of experiments, the effects of CCL2, IL-10, and IL-13 released from the lineage^−^CD34^+^CD31^+^ cells on the suppression for HBD-1 production by NHEK were examined. When NHEK were cultured with fresh media supplemented with 20% (v/v) of culture fluids of lineage^−^CD34^+^CD31^+^ cells, HBD-1 production in these cultures was not demonstrated. However, the antimicrobial peptide was produced in NHEK cultures supplemented with lineage^−^CD34^+^CD31^+^ cell-culture fluids that were previously treated with a mixture of mAbs for IL-10 and CCL2. The suppressor activity of lineage^−^CD34^+^CD31^+^ cell-culture fluids on the peptide production by NHEK was not influenced by anti-IL-13 mAb. These results indicate that IL-10 and CCL2 released from lineage^−^CD34^+^CD31^+^ cells are effectors on the suppression of the peptide production by NHEK. This was confirmed in NHEK cultures supplemented with rIL-10 and/or rCCL2. In the results obtained, HBD-1 production by NHEK was completely inhibited by a mixture of rCCL2 and rIL-10. This indicates that lineage^−^CD34^+^ CD31^+^ cells that appear in association with severe burn injuries are inhibitor cells on HBD-1 production by NHEK. Thus, IL-10 and CCL2 released from lineage^−^CD34^+^CD31^+^ cells are shown to be effectors on their suppressor cell activities.

We have previously reported that immunosuppressive neutrophils (named as PMN-II) are predominantly present in peripheral blood of severely burned patients [24]. Similar to lineage^−^CD34^+^CD31^+^ cells, PMN-II possess the ability to produce CCL2 and IL-10 [24], [36]. These PMN-II that appear in association with severe burn injuries play an important role on the decreased resistance of burn patients to MRSA [36] and *Enterococcus faecalis* infections [37]–[39]. M2 macrophages generated from resident macrophages after stimulation with CCL2 and IL-10 inactivate the host's antibacterial resistance [24], [36]. Thus, M2 macrophages possess minimal bactericidal activities and inhibit macrophage conversion from resident macrophages to M1 macrophages, which are major effector cells against MRSA and enterococcal infections [36]–[39]. Through the production of IL-10 and CCL2, lineage^−^CD34^+^CD31^+^ cells may also be involved in the macrophage conversion from resident macrophages to M2 macrophages.

All severely burned patients, hospitalized at Shriners Hospitals for Children in Galveston, routinely received the standard burn care treatments (physical debridment, irrigation, resuscitation, systemic and topical antibiotic treatment, and aggressive nutrition support). Although some of the blood specimens utilized in this study were obtained from the patients under standard burn care treatments, we do not know how lineage^−^CD34^+^CD31^+^ cell generation is influenced by these treatments. In murine studies, we have previously described the generation of Gr-1^+^CD11b^+^ immature myeloid cells that are corresponding to human lineage^−^CD34^+^CD31^+^ cells [16]. As preliminary studies of these experiments, normal BALB/c mice were exposed to standard burn care treatments (resuscitation, systemic antibiotic treatment and aggressive nutrition support). However, Gr-1^+^CD11b^+^ immature myeloid cells were not isolated from neither the circulation or spleens of these mice. Also, we have isolated Gr-1^+^CD11b^+^ immature myeloid cells from severely burned mice (3^rd^ degree, 30% TBSA burns, 1 to 6 days postburn) [16], [18]. Therefore, now we are thinking that burn injury is the most important factor on the generation of lineage^−^CD34^+^CD31^+^ cells in severely burned patients.

In host antibacterial innate immunities, the importance of β-defensins against bacterial invasions has been well-described [7], [8]. We have demonstrated the deficit of these peptides in tissues surrounding the burn area. Therefore, only 50 CFU/mouse of *Pseudomonas aeruginosa* burn wound infection causes severe systemic infections related to death [18]. The advance of the present study was to determine factors responsible for inhibiting β-defensin production by skin keratinocytes in severely burned patients. CCL2 and IL-10 released from lineage^−^CD34^+^CD31^+^ cells were shown to be key factors for inhibiting β-defensin production by keratinocytes. Further studies on the correlation between the appearance of these cells and clinical outcomes, sepsis, infections and death of burn patients are required.
